# Genome Sequence of a Thermophilic Bacillus, *Geobacillus thermodenitrificans* DSM465

**DOI:** 10.1128/genomeA.01046-13

**Published:** 2013-12-12

**Authors:** Nana Yao, Yi Ren, Wei Wang

**Affiliations:** Key Laboratory of Molecular Microbiology and Technology, Ministry of Education, TEDA School of Biological Sciences and Biotechnology, Nankai University, TEDA, Tianjin, People’s Republic of Chinaa; Tianjin Key Laboratory of Microbial Functional Genomics, Tianjin, People’s Republic of Chinab; School of Food Engineering and Biological Technology, Tianjin University of Science and Technology, Tianjin, People’s Republic of Chinac

## Abstract

*Geobacillus thermodenitrificans* NG80-2 encodes a LadA-mediated alkane degradation pathway, while *G. thermodenitrificans* DSM465 cannot utilize alkanes. Here, we report the draft genome sequence of *G. thermodenitrificans* DSM465, which may help reveal the genomic differences between these two strains in regards to the biodegradation of alkanes.

## GENOME ANNOUNCEMENT

*Geobacillus*, the phylogenetically distinct and physiologically and morphologically consistent taxon of thermophilic bacilli, was recently separated and emended from the genus *Bacillus* (1, 2). Members of *Geobacillus* have been found to live in various kinds of environments and have attracted interest for their potential applications as sources of thermostable enzymes (3). At present, among the 16 strains of *Geobacillus* that have had their genomes sequenced, *Geobacillus thermodenitrificans* strain NG80-2 is the only one of its species. NG80-2 is a thermophilic bacillus capable of LadA (long-chain alkane monooxygenase)-mediated alkane degradation and was isolated from a deep subterranean oil reservoir in northern China (4). However, the type strain of this species, *G. thermodenitrificans* DSM465 (5), isolated from sugar beet juice from extraction installations in Austria, is not a natural alkane degrader. To gain better insight into the genomic difference between DSM465 and NG80-2 in regard to alkane degradation, we report here the draft genome sequence of *G. thermodenitrificans* DSM465.

The genome sequencing of DSM465 was carried out using the Illumina HiSeq 2000 platform at the Majorbio Bio-Pharm Technology Co., Ltd. (Shanghai, China), with the paired-end protocol, followed by *de novo* assembly using SOAP*denovo* (http://soap.genomics.org.cn/soapdenovo.html). Protein-encoding genes, rRNA operons, and tRNAs were predicted by Glimmer 3.0 (6), RNAmmer (7), and tRNAscan-SE (8), respectively. Functional annotation was based on BLASTp with the KEGG, Swiss-Prot, COG, and nonredundant (NR) databases. A total of 5,358,680 filtered paired-end reads were generated, with a mean length of 91 bp, corresponding to 307-fold coverage of the genome. Eighty-eight contigs contained in 72 scaffolds were generated, with an N_50_ of 264,274 bp. The final genome draft of DSM465 consists of 3,402,060 bp, with a G+C content of 49.05%, which was identical to that of NG80-2, and 3,443 protein-encoding genes were predicted in total.

In NG80-2, alkanes are first converted to the corresponding fatty acids by LadA, alcohol dehydrogenase (ADH), and aldehyde dehydrogenase (ALDH) before entering the β-oxidation pathway as the coenzyme A (CoA)-activated form (fatty acid CoA) to be further degraded (4). Comparative genomic analysis showed that 2,991 genes are shared by NG80-2 and DSM465 and 415 and 439 genes are unique to NG80-2 and DSM465, respectively. As expected, *ladA* (*GTNG_3499*), located on the plasmid of NG80-2, was not found in the DSM465 genome. Conversely, genes for the other enzymes involved in the alkane degradation pathway, including ADHs (*GTNG_1754* and *GTNG_2878*) (9), ALDHs (*GTNG_3117*) (10), and fatty acid CoA ligases (*GTNG_0892* and *GTNG_1447*) (11), and the proteins responsible for the β-oxidation pathway were all found in DSM465. Deep analysis to interpret this mechanism of LadA-mediated alkane degradation is being carried out, which may provide us more information to understand bacterial evolution under environment pressure.

### Nucleotide sequence accession numbers.

The whole-genome shotgun project for *G. thermodenitrificans* DSM465 has been deposited at DDBJ/EMBL/GenBank under the accession no. AYKT00000000. The version described in this paper is the first version, AYKT01000000.
